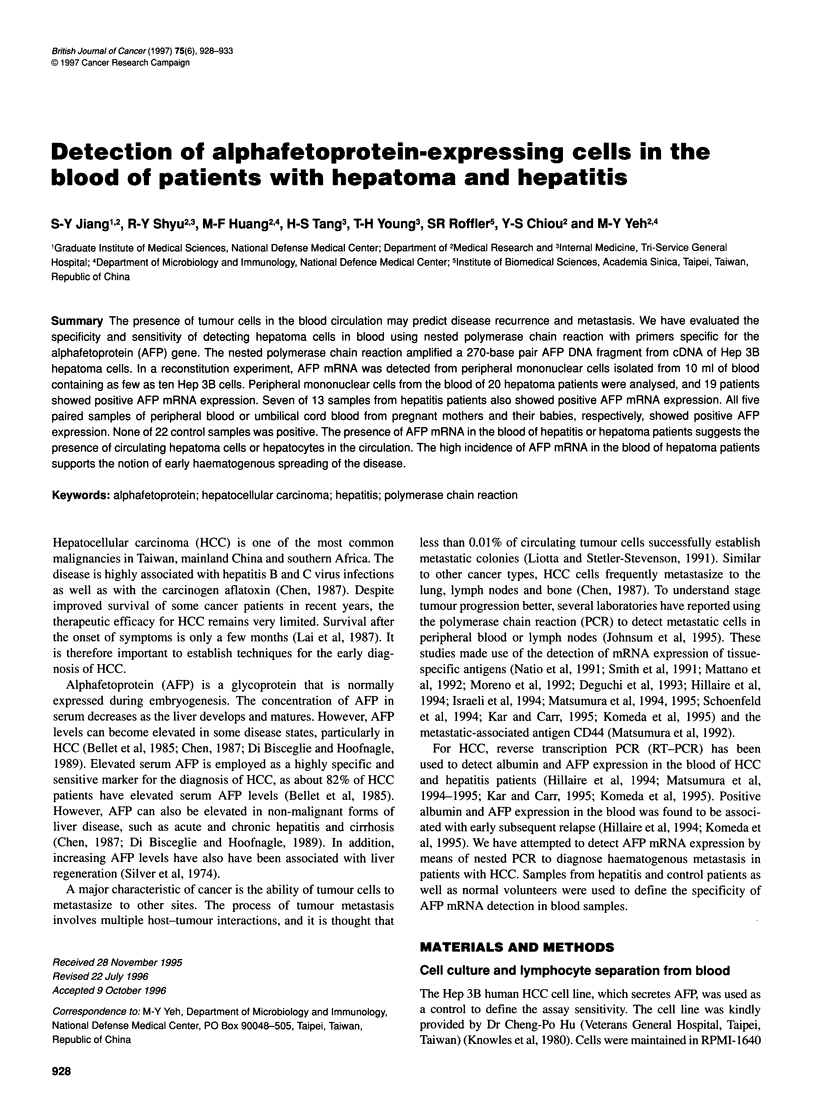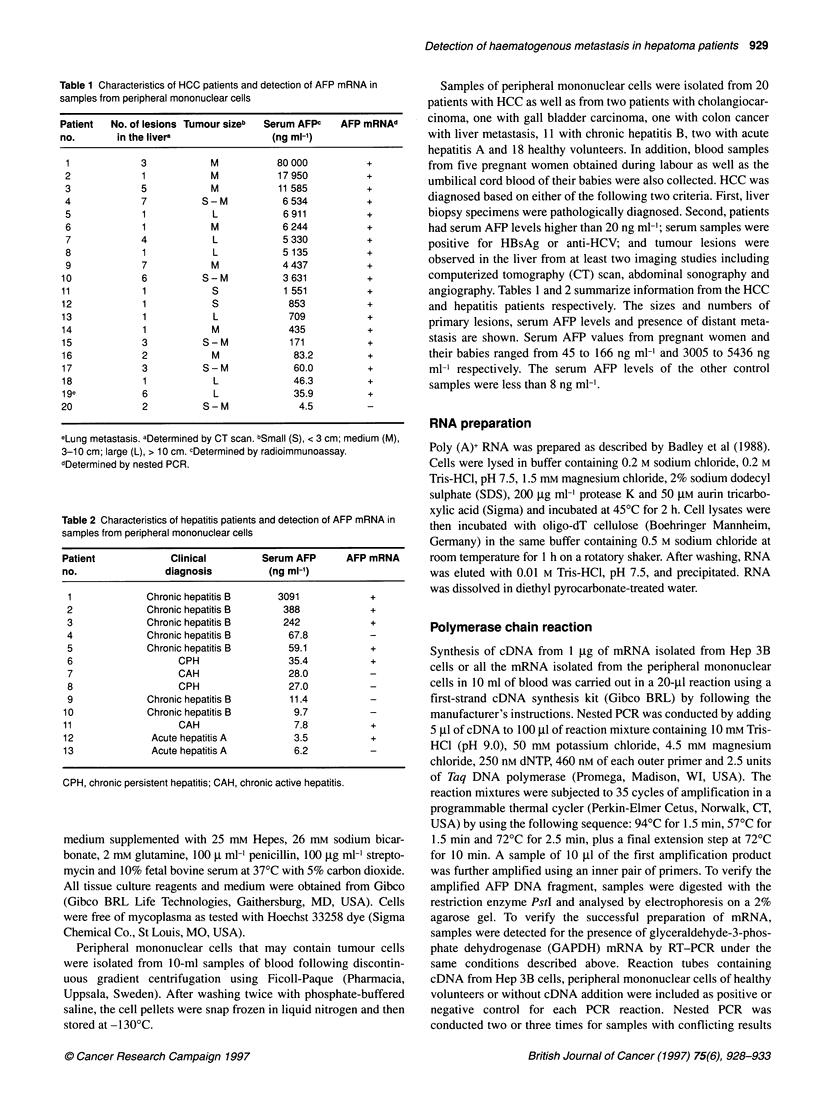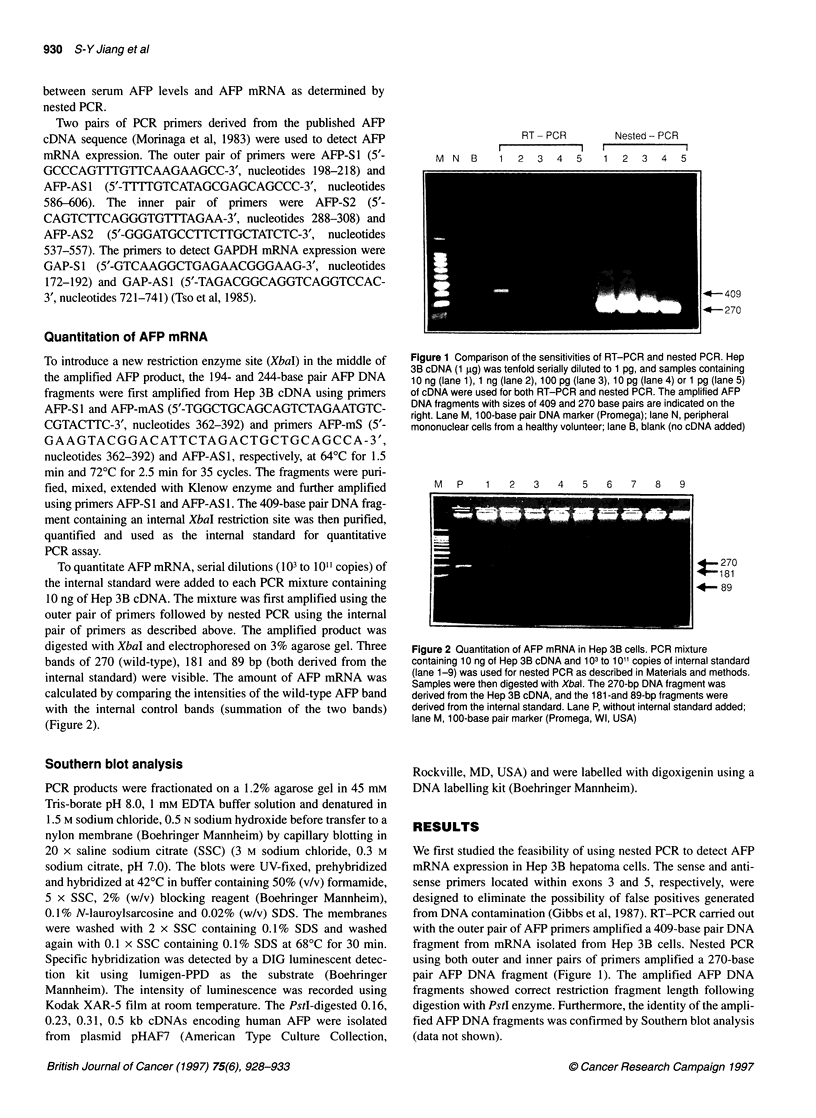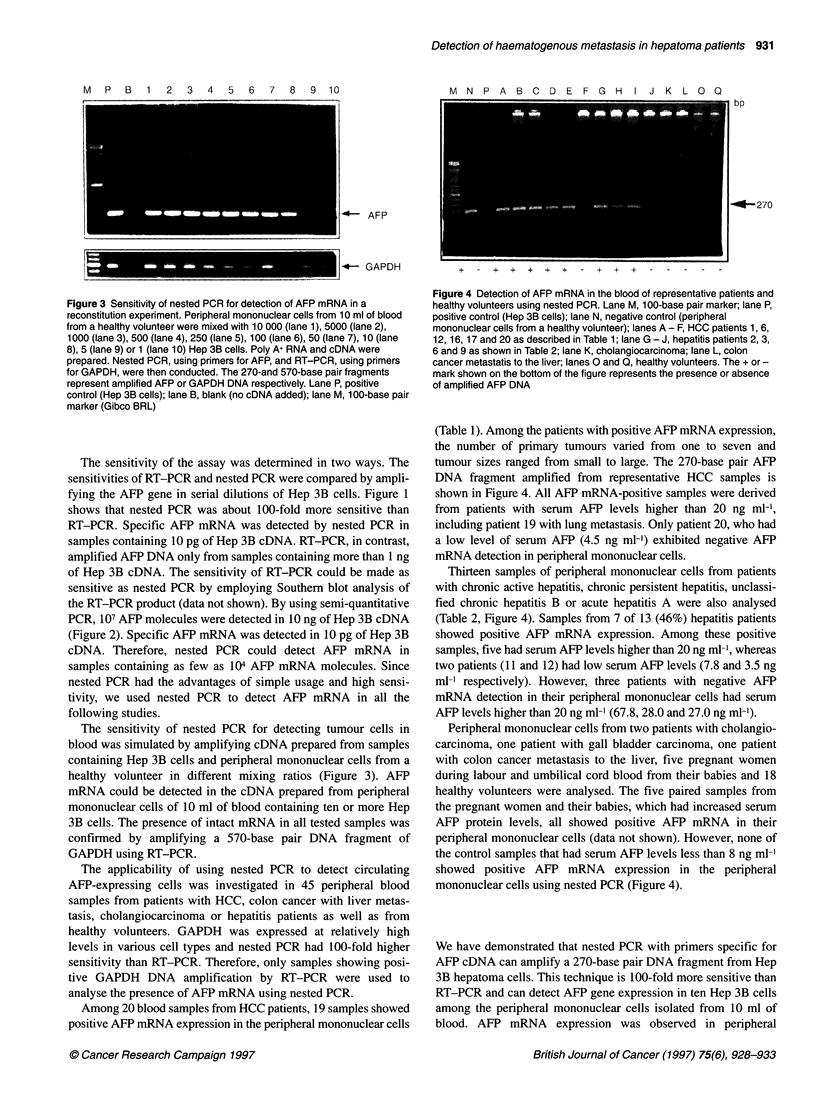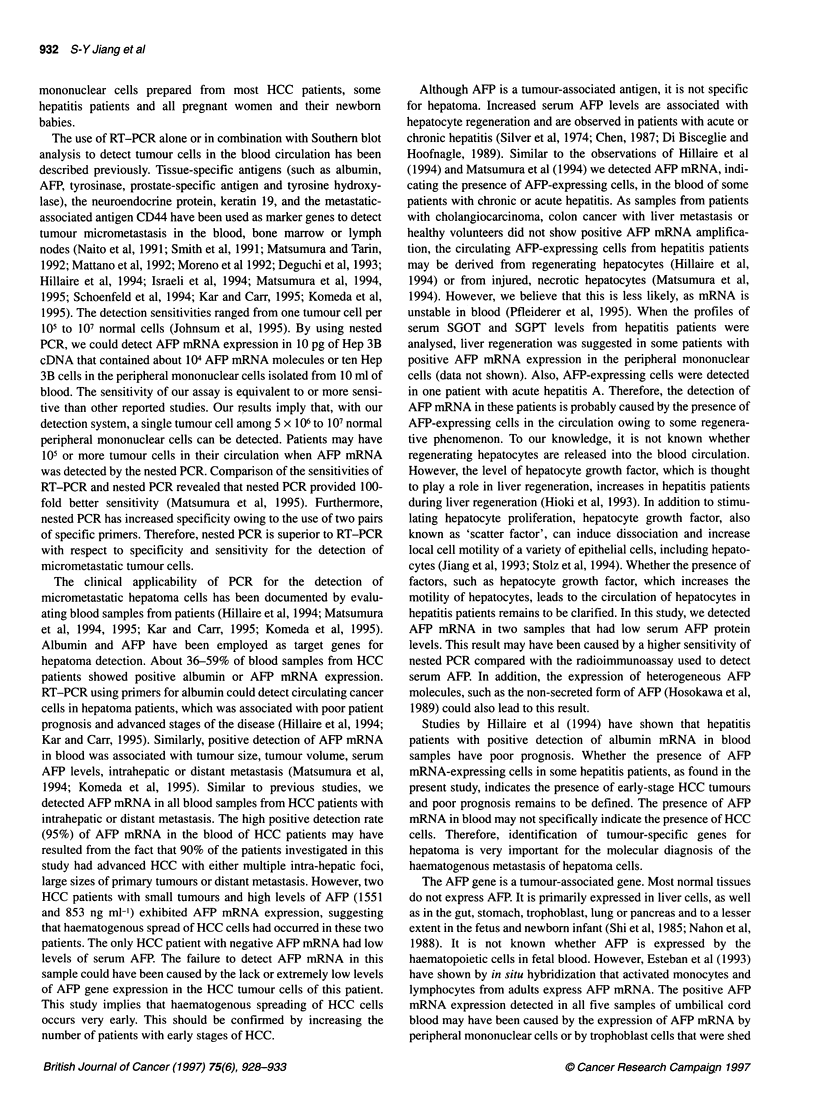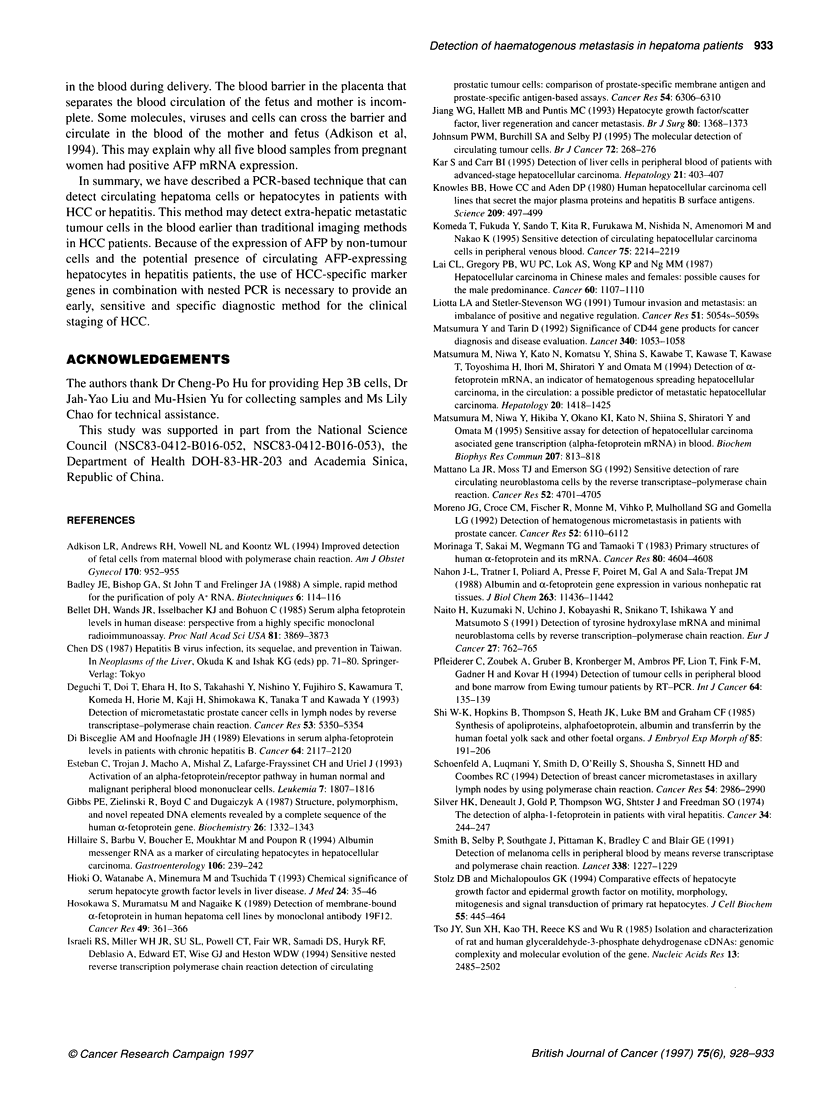# Detection of alphafetoprotein-expressing cells in the blood of patients with hepatoma and hepatitis.

**DOI:** 10.1038/bjc.1997.163

**Published:** 1997

**Authors:** S. Y. Jiang, R. Y. Shyu, M. F. Huang, H. S. Tang, T. H. Young, S. R. Roffler, Y. S. Chiou, M. Y. Yeh

**Affiliations:** Graduate Institute of Medical Sciences, National Defense Medical Center, Taipei, Taiwan, Republic of China.

## Abstract

**Images:**


British Journal of Cancer (1997) **75**(6), 928–933
© 1997 Cancer Research Campaign

### Detection of alphafetoprotein-expressing cells in the blood of patients with hepatoma and hepatitis

**S-Y Jiang[1,2], R-Y Shyu[2,3], M-F Huang[2,4], H-S Tang[3], T-H Young[3], SR Roffler[5], Y-S Chiou[2] and M-Y Yeh[2,4]**

[1]Graduate Institute of Medical Sciences, National Defense Medical Center; Department of [2]Medical Research and [3]Internal Medicine, Tri-Service General Hospital; [4]Department of Microbiology and Immunology, National Defence Medical Center; [5]Institute of Biomedical Sciences, Academia Sinica, Taipei, Taiwan, Republic of China

**Summary** The presence of tumour cells in the blood circulation may predict disease recurrence and metastasis. We have evaluated the specificity and sensitivity of detecting hepatoma cells in blood using nested polymerase chain reaction with primers specific for the alphafetoprotein (AFP) gene. The nested polymerase chain reaction amplified a 270-base pair AFP DNA fragment from cDNA of Hep 3B hepatoma cells. In a reconstitution experiment, AFP mRNA was detected from peripheral mononuclear cells isolated from 10 ml of blood containing as few as ten Hep 3B cells. Peripheral mononuclear cells from the blood of 20 hepatoma patients were analysed, and 19 patients showed positive AFP mRNA expression. Seven of 13 samples from hepatitis patients also showed positive AFP mRNA expression. All five paired samples of peripheral blood or umbilical cord blood from pregnant mothers and their babies, respectively, showed positive AFP expression. None of 22 control samples was positive. The presence of AFP mRNA in the blood of hepatitis or hepatoma patients suggests the presence of circulating hepatoma cells or hepatocytes in the circulation. The high incidence of AFP mRNA in the blood of hepatoma patients supports the notion of early haematogenous spreading of the disease.

**Keywords:** alphafetoprotein; hepatocellular carcinoma; hepatitis; polymerase chain reaction

Hepatocellular carcinoma (HCC) is one of the most common malignancies in Taiwan, mainland China and southern Africa. The disease is highly associated with hepatitis B and C virus infections as well as with the carcinogen aflatoxin (Chen, 1987). Despite improved survival of some cancer patients in recent years, the therapeutic efficacy for HCC remains very limited. Survival after the onset of symptoms is only a few months (Lai et al, 1987). It is therefore important to establish techniques for the early diagnosis of HCC.

Alphafetoprotein (AFP) is a glycoprotein that is normally expressed during embryogenesis. The concentration of AFP in serum decreases as the liver develops and matures. However, AFP levels can become elevated in some disease states, particularly in HCC (Bellet et al, 1985; Chen, 1987; Di Bisceglie and Hoofnagle, 1989). Elevated serum AFP is employed as a highly specific and sensitive marker for the diagnosis of HCC, as about 82% of HCC patients have elevated serum AFP levels (Bellet et al, 1985). However, AFP can also be elevated in non-malignant forms of liver disease, such as acute and chronic hepatitis and cirrhosis (Chen, 1987; Di Bisceglie and Hoofnagle, 1989). In addition, increasing AFP levels have also have been associated with liver regeneration (Silver et al, 1974).

A major characteristic of cancer is the ability of tumour cells to metastasize to other sites. The process of tumour metastasis involves multiple host–tumour interactions, and it is thought that less than 0.01% of circulating tumour cells successfully establish metastatic colonies (Liotta and Stetler-Stevenson, 1991). Similar to other cancer types, HCC cells frequently metastasize to the lung, lymph nodes and bone (Chen, 1987). To understand stage tumour progression better, several laboratories have reported using the polymerase chain reaction (PCR) to detect metastatic cells in peripheral blood or lymph nodes (Johnsum et al, 1995). These studies made use of the detection of mRNA expression of tissue-specific antigens (Natio et al, 1991; Smith et al, 1991; Mattano et al, 1992; Moreno et al, 1992; Deguchi et al, 1993; Hillaire et al, 1994; Israeli et al, 1994; Matsumura et al, 1994, 1995; Schoenfeld et al, 1994; Kar and Carr, 1995; Komeda et al, 1995) and the metastatic-associated antigen CD44 (Matsumura et al, 1992).

For HCC, reverse transcription PCR (RT–PCR) has been used to detect albumin and AFP expression in the blood of HCC and hepatitis patients (Hillaire et al, 1994; Matsumura et al, 1994–1995; Kar and Carr, 1995; Komeda et al, 1995). Positive albumin and AFP expression in the blood was found to be associated with early subsequent relapse (Hillaire et al, 1994; Komeda et al, 1995). We have attempted to detect AFP mRNA expression by means of nested PCR to diagnose haematogenous metastasis in patients with HCC. Samples from hepatitis and control patients as well as normal volunteers were used to define the specificity of AFP mRNA detection in blood samples.

### MATERIALS AND METHODS

### Cell culture and lymphocyte separation from blood

The Hep 3B human HCC cell line, which secretes AFP, was used as a control to define the assay sensitivity. The cell line was kindly provided by Dr Cheng-Po Hu (Veterans General Hospital, Taipei, Taiwan) (Knowles et al, 1980). Cells were maintained in RPMI-1640

*Received 28 November 1995*
*Revised 22 July 1996*
*Accepted 9 October 1996*

*Correspondence to:* M-Y Yeh, Department of Microbiology and Immunology, National Defense Medical Center, PO Box 90048–505, Taipei, Taiwan, Republic of China

**Table 1** Characteristics of HCC patients and detection of AFP mRNA in samples from peripheral mononuclear cells

| Patient no. | No. of lesions in the liver[a] | Tumour size[b] | Serum AFP[c] (ng ml$^{-1}$) | AFP mRNA[d] |
|---|---|---|---|---|
| 1 | 3 | M | 80 000 | + |
| 2 | 1 | M | 17 950 | + |
| 3 | 5 | M | 11 585 | + |
| 4 | 7 | S–M | 6 534 | + |
| 5 | 1 | L | 6 911 | + |
| 6 | 1 | M | 6 244 | + |
| 7 | 4 | L | 5 330 | + |
| 8 | 1 | L | 5 135 | + |
| 9 | 7 | M | 4 437 | + |
| 10 | 6 | S–M | 3 631 | + |
| 11 | 1 | S | 1 551 | + |
| 12 | 1 | S | 853 | + |
| 13 | 1 | L | 709 | + |
| 14 | 1 | M | 435 | + |
| 15 | 3 | S–M | 171 | + |
| 16 | 2 | M | 83.2 | + |
| 17 | 3 | S–M | 60.0 | + |
| 18 | 1 | L | 46.3 | + |
| 19[e] | 6 | L | 35.9 | + |
| 20 | 2 | S–M | 4.5 | – |

[e]Lung metastasis. [a]Determined by CT scan. [b]Small (S), < 3 cm; medium (M), 3–10 cm; large (L), > 10 cm. [c]Determined by radioimmunoassay. [d]Determined by nested PCR.

**Table 2** Characteristics of hepatitis patients and detection of AFP mRNA in samples from peripheral mononuclear cells

| Patient no. | Clinical diagnosis | Serum AFP (ng ml$^{-1}$) | AFP mRNA |
|---|---|---|---|
| 1 | Chronic hepatitis B | 3091 | + |
| 2 | Chronic hepatitis B | 388 | + |
| 3 | Chronic hepatitis B | 242 | + |
| 4 | Chronic hepatitis B | 67.8 | – |
| 5 | Chronic hepatitis B | 59.1 | + |
| 6 | CPH | 35.4 | + |
| 7 | CAH | 28.0 | – |
| 8 | CPH | 27.0 | – |
| 9 | Chronic hepatitis B | 11.4 | – |
| 10 | Chronic hepatitis B | 9.7 | – |
| 11 | CAH | 7.8 | + |
| 12 | Acute hepatitis A | 3.5 | + |
| 13 | Acute hepatitis A | 6.2 | – |

CPH, chronic persistent hepatitis; CAH, chronic active hepatitis.

medium supplemented with 25 mM Hepes, 26 mM sodium bicarbonate, 2 mM glutamine, 100 μ ml$^{-1}$ penicillin, 100 μg ml$^{-1}$ streptomycin and 10% fetal bovine serum at 37°C with 5% carbon dioxide. All tissue culture reagents and medium were obtained from Gibco (Gibco BRL Life Technologies, Gaithersburg, MD, USA). Cells were free of mycoplasma as tested with Hoechst 33258 dye (Sigma Chemical Co., St Louis, MO, USA).

Peripheral mononuclear cells that may contain tumour cells were isolated from 10-ml samples of blood following discontinuous gradient centrifugation using Ficoll-Paque (Pharmacia, Uppsala, Sweden). After washing twice with phosphate-buffered saline, the cell pellets were snap frozen in liquid nitrogen and then stored at –130°C.

Samples of peripheral mononuclear cells were isolated from 20 patients with HCC as well as from two patients with cholangiocarcinoma, one with gall bladder carcinoma, one with colon cancer with liver metastasis, 11 with chronic hepatitis B, two with acute hepatitis A and 18 healthy volunteers. In addition, blood samples from five pregnant women obtained during labour as well as the umbilical cord blood of their babies were also collected. HCC was diagnosed based on either of the following two criteria. First, liver biopsy specimens were pathologically diagnosed. Second, patients had serum AFP levels higher than 20 ng ml$^{-1}$; serum samples were positive for HBsAg or anti-HCV; and tumour lesions were observed in the liver from at least two imaging studies including computerized tomography (CT) scan, abdominal sonography and angiography. Tables 1 and 2 summarize information from the HCC and hepatitis patients respectively. The sizes and numbers of primary lesions, serum AFP levels and presence of distant metastasis are shown. Serum AFP values from pregnant women and their babies ranged from 45 to 166 ng ml$^{-1}$ and 3005 to 5436 ng ml$^{-1}$ respectively. The serum AFP levels of the other control samples were less than 8 ng ml$^{-1}$.

### RNA preparation

Poly (A)$^+$ RNA was prepared as described by Badley et al (1988). Cells were lysed in buffer containing 0.2 M sodium chloride, 0.2 M Tris-HCl, pH 7.5, 1.5 mM magnesium chloride, 2% sodium dodecyl sulphate (SDS), 200 μg ml$^{-1}$ protease K and 50 μM aurin tricarboxylic acid (Sigma) and incubated at 45°C for 2 h. Cell lysates were then incubated with oligo-dT cellulose (Boehringer Mannheim, Germany) in the same buffer containing 0.5 M sodium chloride at room temperature for 1 h on a rotatory shaker. After washing, RNA was eluted with 0.01 M Tris-HCl, pH 7.5, and precipitated. RNA was dissolved in diethyl pyrocarbonate-treated water.

### Polymerase chain reaction

Synthesis of cDNA from 1 μg of mRNA isolated from Hep 3B cells or all the mRNA isolated from the peripheral mononuclear cells in 10 ml of blood was carried out in a 20-μl reaction using a first-strand cDNA synthesis kit (Gibco BRL) by following the manufacturer's instructions. Nested PCR was conducted by adding 5 μl of cDNA to 100 μl of reaction mixture containing 10 mM Tris-HCl (pH 9.0), 50 mM potassium chloride, 4.5 mM magnesium chloride, 250 nM dNTP, 460 nM of each outer primer and 2.5 units of *Taq* DNA polymerase (Promega, Madison, WI, USA). The reaction mixtures were subjected to 35 cycles of amplification in a programmable thermal cycler (Perkin-Elmer Cetus, Norwalk, CT, USA) by using the following sequence: 94°C for 1.5 min, 57°C for 1.5 min and 72°C for 2.5 min, plus a final extension step at 72°C for 10 min. A sample of 10 μl of the first amplification product was further amplified using an inner pair of primers. To verify the amplified AFP DNA fragment, samples were digested with the restriction enzyme *Pst*I and analysed by electrophoresis on a 2% agarose gel. To verify the successful preparation of mRNA, samples were detected for the presence of glyceraldehyde-3-phosphate dehydrogenase (GAPDH) mRNA by RT–PCR under the same conditions described above. Reaction tubes containing cDNA from Hep 3B cells, peripheral mononuclear cells of healthy volunteers or without cDNA addition were included as positive or negative control for each PCR reaction. Nested PCR was conducted two or three times for samples with conflicting results

© Cancer Research Campaign 1997

between serum AFP levels and AFP mRNA as determined by nested PCR.

Two pairs of PCR primers derived from the published AFP cDNA sequence (Morinaga et al, 1983) were used to detect AFP mRNA expression. The outer pair of primers were AFP-S1 (5′-GCCCAGTTTGTTCAAGAAGCC-3′, nucleotides 198–218) and AFP-AS1 (5′-TTTTGTCATAGCGAGCAGCCC-3′, nucleotides 586–606). The inner pair of primers were AFP-S2 (5′-CAGTCTTCAGGGTGTTTAGAA-3′, nucleotides 288–308) and AFP-AS2 (5′-GGGATGCCTTCTTGCTATCTC-3′, nucleotides 537–557). The primers to detect GAPDH mRNA expression were GAP-S1 (5′-GTCAAGGCTGAGAACGGGAAG-3′, nucleotides 172–192) and GAP-AS1 (5′-TAGACGGCAGGTCAGGTCCAC-3′, nucleotides 721–741) (Tso et al, 1985).

**Figure 1** Comparison of the sensitivities of RT–PCR and nested PCR. Hep 3B cDNA (1 μg) was tenfold serially diluted to 1 pg, and samples containing 10 ng (lane 1), 1 ng (lane 2), 100 pg (lane 3), 10 pg (lane 4) or 1 pg (lane 5) of cDNA were used for both RT–PCR and nested PCR. The amplified AFP DNA fragments with sizes of 409 and 270 base pairs are indicated on the right. Lane M, 100-base pair DNA marker (Promega); lane N, peripheral mononuclear cells from a healthy volunteer; lane B, blank (no cDNA added)

### Quantitation of AFP mRNA

To introduce a new restriction enzyme site (*Xba*I) in the middle of the amplified AFP product, the 194- and 244-base pair AFP DNA fragments were first amplified from Hep 3B cDNA using primers AFP-S1 and AFP-mAS (5′-TGGCTGCAGCAGTCTAGAATGTC-CGTACTTC-3′, nucleotides 362–392) and primers AFP-mS (5′-GAAGTACGGACATTCTAGACTGCTGCAGCCA-3′, nucleotides 362–392) and AFP-AS1, respectively, at 64°C for 1.5 min and 72°C for 2.5 min for 35 cycles. The fragments were purified, mixed, extended with Klenow enzyme and further amplified using primers AFP-S1 and AFP-AS1. The 409-base pair DNA fragment containing an internal *Xba*I restriction site was then purified, quantified and used as the internal standard for quantitative PCR assay.

To quantitate AFP mRNA, serial dilutions ($10^3$ to $10^{11}$ copies) of the internal standard were added to each PCR mixture containing 10 ng of Hep 3B cDNA. The mixture was first amplified using the outer pair of primers followed by nested PCR using the internal pair of primers as described above. The amplified product was digested with *Xba*I and electrophoresed on 3% agarose gel. Three bands of 270 (wild-type), 181 and 89 bp (both derived from the internal standard) were visible. The amount of AFP mRNA was calculated by comparing the intensities of the wild-type AFP band with the internal control bands (summation of the two bands) (Figure 2).

**Figure 2** Quantitation of AFP mRNA in Hep 3B cells. PCR mixture containing 10 ng of Hep 3B cDNA and $10^3$ to $10^{11}$ copies of internal standard (lane 1–9) was used for nested PCR as described in Materials and methods. Samples were then digested with *Xba*I. The 270-bp DNA fragment was derived from the Hep 3B cDNA, and the 181-and 89-bp fragments were derived from the internal standard. Lane P, without internal standard added; lane M, 100-base pair marker (Promega, WI, USA)

### Southern blot analysis

PCR products were fractionated on a 1.2% agarose gel in 45 mM Tris-borate pH 8.0, 1 mM EDTA buffer solution and denatured in 1.5 M sodium chloride, 0.5 N sodium hydroxide before transfer to a nylon membrane (Boehringer Mannheim) by capillary blotting in 20 × saline sodium citrate (SSC) (3 M sodium chloride, 0.3 M sodium citrate, pH 7.0). The blots were UV-fixed, prehybridized and hybridized at 42°C in buffer containing 50% (v/v) formamide, 5 × SSC, 2% (w/v) blocking reagent (Boehringer Mannheim), 0.1% *N*-lauroylsarcosine and 0.02% (w/v) SDS. The membranes were washed with 2 × SSC containing 0.1% SDS and washed again with 0.1 × SSC containing 0.1% SDS at 68°C for 30 min. Specific hybridization was detected by a DIG luminescent detection kit using lumigen-PPD as the substrate (Boehringer Mannheim). The intensity of luminescence was recorded using Kodak XAR-5 film at room temperature. The *Pst*I-digested 0.16, 0.23, 0.31, 0.5 kb cDNAs encoding human AFP were isolated from plasmid pHAF7 (American Type Culture Collection, Rockville, MD, USA) and were labelled with digoxigenin using a DNA labelling kit (Boehringer Mannheim).

### RESULTS

We first studied the feasibility of using nested PCR to detect AFP mRNA expression in Hep 3B hepatoma cells. The sense and antisense primers located within exons 3 and 5, respectively, were designed to eliminate the possibility of false positives generated from DNA contamination (Gibbs et al, 1987). RT–PCR carried out with the outer pair of AFP primers amplified a 409-base pair DNA fragment from mRNA isolated from Hep 3B cells. Nested PCR using both outer and inner pairs of primers amplified a 270-base pair AFP DNA fragment (Figure 1). The amplified AFP DNA fragments showed correct restriction fragment length following digestion with *Pst*I enzyme. Furthermore, the identity of the amplified AFP DNA fragments was confirmed by Southern blot analysis (data not shown).

© Cancer Research Campaign 1997

**Figure 3** Sensitivity of nested PCR for detection of AFP mRNA in a reconstitution experiment. Peripheral mononuclear cells from 10 ml of blood from a healthy volunteer were mixed with 10 000 (lane 1), 5000 (lane 2), 1000 (lane 3), 500 (lane 4), 250 (lane 5), 100 (lane 6), 50 (lane 7), 10 (lane 8), 5 (lane 9) or 1 (lane 10) Hep 3B cells. Poly A+ RNA and cDNA were prepared. Nested PCR, using primers for AFP, and RT–PCR, using primers for GAPDH, were then conducted. The 270-and 570-base pair fragments represent amplified AFP or GAPDH DNA respectively. Lane P, positive control (Hep 3B cells); lane B, blank (no cDNA added); lane M, 100-base pair marker (Gibco BRL)

**Figure 4** Detection of AFP mRNA in the blood of representative patients and healthy volunteers using nested PCR. Lane M, 100-base pair marker; lane P, positive control (Hep 3B cells); lane N, negative control (peripheral mononuclear cells from a healthy volunteer); lanes A – F, HCC patients 1, 6, 12, 16, 17 and 20 as described in Table 1; lane G – J, hepatitis patients 2, 3, 6 and 9 as shown in Table 2; lane K, cholangiocarcinoma; lane L, colon cancer metastatis to the liver; lanes O and Q, healthy volunteers. The + or – mark shown on the bottom of the figure represents the presence or absence of amplified AFP DNA

The sensitivity of the assay was determined in two ways. The sensitivities of RT–PCR and nested PCR were compared by amplifying the AFP gene in serial dilutions of Hep 3B cells. Figure 1 shows that nested PCR was about 100-fold more sensitive than RT–PCR. Specific AFP mRNA was detected by nested PCR in samples containing 10 pg of Hep 3B cDNA. RT–PCR, in contrast, amplified AFP DNA only from samples containing more than 1 ng of Hep 3B cDNA. The sensitivity of RT–PCR could be made as sensitive as nested PCR by employing Southern blot analysis of the RT–PCR product (data not shown). By using semi-quantitative PCR, $10^7$ AFP molecules were detected in 10 ng of Hep 3B cDNA (Figure 2). Specific AFP mRNA was detected in 10 pg of Hep 3B cDNA. Therefore, nested PCR could detect AFP mRNA in samples containing as few as $10^4$ AFP mRNA molecules. Since nested PCR had the advantages of simple usage and high sensitivity, we used nested PCR to detect AFP mRNA in all the following studies.

The sensitivity of nested PCR for detecting tumour cells in blood was simulated by amplifying cDNA prepared from samples containing Hep 3B cells and peripheral mononuclear cells from a healthy volunteer in different mixing ratios (Figure 3). AFP mRNA could be detected in the cDNA prepared from peripheral mononuclear cells of 10 ml of blood containing ten or more Hep 3B cells. The presence of intact mRNA in all tested samples was confirmed by amplifying a 570-base pair DNA fragment of GAPDH using RT–PCR.

The applicability of using nested PCR to detect circulating AFP-expressing cells was investigated in 45 peripheral blood samples from patients with HCC, colon cancer with liver metastasis, cholangiocarcinoma or hepatitis patients as well as from healthy volunteers. GAPDH was expressed at relatively high levels in various cell types and nested PCR had 100-fold higher sensitivity than RT–PCR. Therefore, only samples showing positive GAPDH DNA amplification by RT–PCR were used to analyse the presence of AFP mRNA using nested PCR.

Among 20 blood samples from HCC patients, 19 samples showed positive AFP mRNA expression in the peripheral mononuclear cells (Table 1). Among the patients with positive AFP mRNA expression, the number of primary tumours varied from one to seven and tumour sizes ranged from small to large. The 270-base pair AFP DNA fragment amplified from representative HCC samples is shown in Figure 4. All AFP mRNA-positive samples were derived from patients with serum AFP levels higher than 20 ng $ml^{-1}$, including patient 19 with lung metastasis. Only patient 20, who had a low level of serum AFP (4.5 ng $ml^{-1}$) exhibited negative AFP mRNA detection in peripheral mononuclear cells.

Thirteen samples of peripheral mononuclear cells from patients with chronic active hepatitis, chronic persistent hepatitis, unclassified chronic hepatitis B or acute hepatitis A were also analysed (Table 2, Figure 4). Samples from 7 of 13 (46%) hepatitis patients showed positive AFP mRNA expression. Among these positive samples, five had serum AFP levels higher than 20 ng $ml^{-1}$, whereas two patients (11 and 12) had low serum AFP levels (7.8 and 3.5 ng $ml^{-1}$ respectively). However, three patients with negative AFP mRNA detection in their peripheral mononuclear cells had serum AFP levels higher than 20 ng $ml^{-1}$ (67.8, 28.0 and 27.0 ng $ml^{-1}$).

Peripheral mononuclear cells from two patients with cholangiocarcinoma, one patient with gall bladder carcinoma, one patient with colon cancer metastasis to the liver, five pregnant women during labour and umbilical cord blood from their babies and 18 healthy volunteers were analysed. The five paired samples from the pregnant women and their babies, which had increased serum AFP protein levels, all showed positive AFP mRNA in their peripheral mononuclear cells (data not shown). However, none of the control samples that had serum AFP levels less than 8 ng $ml^{-1}$ showed positive AFP mRNA expression in the peripheral mononuclear cells using nested PCR (Figure 4).

We have demonstrated that nested PCR with primers specific for AFP cDNA can amplify a 270-base pair DNA fragment from Hep 3B hepatoma cells. This technique is 100-fold more sensitive than RT–PCR and can detect AFP gene expression in ten Hep 3B cells among the peripheral mononuclear cells isolated from 10 ml of blood. AFP mRNA expression was observed in peripheral

© Cancer Research Campaign 1997 

mononuclear cells prepared from most HCC patients, some hepatitis patients and all pregnant women and their newborn babies.

The use of RT–PCR alone or in combination with Southern blot analysis to detect tumour cells in the blood circulation has been described previously. Tissue-specific antigens (such as albumin, AFP, tyrosinase, prostate-specific antigen and tyrosine hydroxylase), the neuroendocrine protein, keratin 19, and the metastatic-associated antigen CD44 have been used as marker genes to detect tumour micrometastasis in the blood, bone marrow or lymph nodes (Naito et al, 1991; Smith et al, 1991; Matsumura and Tarin, 1992; Mattano et al, 1992; Moreno et al 1992; Deguchi et al, 1993; Hillaire et al, 1994; Israeli et al, 1994; Matsumura et al, 1994, 1995; Schoenfeld et al, 1994; Kar and Carr, 1995; Komeda et al, 1995). The detection sensitivities ranged from one tumour cell per $10^5$ to $10^7$ normal cells (Johnsum et al, 1995). By using nested PCR, we could detect AFP mRNA expression in 10 pg of Hep 3B cDNA that contained about $10^4$ AFP mRNA molecules or ten Hep 3B cells in the peripheral mononuclear cells isolated from 10 ml of blood. The sensitivity of our assay is equivalent to or more sensitive than other reported studies. Our results imply that, with our detection system, a single tumour cell among $5 \times 10^6$ to $10^7$ normal peripheral mononuclear cells can be detected. Patients may have $10^5$ or more tumour cells in their circulation when AFP mRNA was detected by the nested PCR. Comparison of the sensitivities of RT–PCR and nested PCR revealed that nested PCR provided 100-fold better sensitivity (Matsumura et al, 1995). Furthermore, nested PCR has increased specificity owing to the use of two pairs of specific primers. Therefore, nested PCR is superior to RT–PCR with respect to specificity and sensitivity for the detection of micrometastatic tumour cells.

The clinical applicability of PCR for the detection of micrometastatic hepatoma cells has been documented by evaluating blood samples from patients (Hillaire et al, 1994; Matsumura et al, 1994, 1995; Kar and Carr, 1995; Komeda et al, 1995). Albumin and AFP have been employed as target genes for hepatoma detection. About 36–59% of blood samples from HCC patients showed positive albumin or AFP mRNA expression. RT–PCR using primers for albumin could detect circulating cancer cells in hepatoma patients, which was associated with poor patient prognosis and advanced stages of the disease (Hillaire et al, 1994; Kar and Carr, 1995). Similarly, positive detection of AFP mRNA in blood was associated with tumour size, tumour volume, serum AFP levels, intrahepatic or distant metastasis (Matsumura et al, 1994; Komeda et al, 1995). Similar to previous studies, we detected AFP mRNA in all blood samples from HCC patients with intrahepatic or distant metastasis. The high positive detection rate (95%) of AFP mRNA in the blood of HCC patients may have resulted from the fact that 90% of the patients investigated in this study had advanced HCC with either multiple intra-hepatic foci, large sizes of primary tumours or distant metastasis. However, two HCC patients with small tumours and high levels of AFP (1551 and 853 ng $ml^{-1}$) exhibited AFP mRNA expression, suggesting that haematogenous spread of HCC cells had occurred in these two patients. The only HCC patient with negative AFP mRNA had low levels of serum AFP. The failure to detect AFP mRNA in this sample could have been caused by the lack or extremely low levels of AFP gene expression in the HCC tumour cells of this patient. This study implies that haematogenous spreading of HCC cells occurs very early. This should be confirmed by increasing the number of patients with early stages of HCC.

Although AFP is a tumour-associated antigen, it is not specific for hepatoma. Increased serum AFP levels are associated with hepatocyte regeneration and are observed in patients with acute or chronic hepatitis (Silver et al, 1974; Chen, 1987; Di Bisceglie and Hoofnagle, 1989). Similar to the observations of Hillaire et al (1994) and Matsumura et al (1994) we detected AFP mRNA, indicating the presence of AFP-expressing cells, in the blood of some patients with chronic or acute hepatitis. As samples from patients with cholangiocarcinoma, colon cancer with liver metastasis or healthy volunteers did not show positive AFP mRNA amplification, the circulating AFP-expressing cells from hepatitis patients may be derived from regenerating hepatocytes (Hillaire et al, 1994) or from injured, necrotic hepatocytes (Matsumura et al, 1994). However, we believe that this is less likely, as mRNA is unstable in blood (Pfleiderer et al, 1995). When the profiles of serum SGOT and SGPT levels from hepatitis patients were analysed, liver regeneration was suggested in some patients with positive AFP mRNA expression in the peripheral mononuclear cells (data not shown). Also, AFP-expressing cells were detected in one patient with acute hepatitis A. Therefore, the detection of AFP mRNA in these patients is probably caused by the presence of AFP-expressing cells in the circulation owing to some regenerative phenomenon. To our knowledge, it is not known whether regenerating hepatocytes are released into the blood circulation. However, the level of hepatocyte growth factor, which is thought to play a role in liver regeneration, increases in hepatitis patients during liver regeneration (Hioki et al, 1993). In addition to stimulating hepatocyte proliferation, hepatocyte growth factor, also known as 'scatter factor', can induce dissociation and increase local cell motility of a variety of epithelial cells, including hepatocytes (Jiang et al, 1993; Stolz et al, 1994). Whether the presence of factors, such as hepatocyte growth factor, which increases the motility of hepatocytes, leads to the circulation of hepatocytes in hepatitis patients remains to be clarified. In this study, we detected AFP mRNA in two samples that had low serum AFP protein levels. This result may have been caused by a higher sensitivity of nested PCR compared with the radioimmunoassay used to detect serum AFP. In addition, the expression of heterogeneous AFP molecules, such as the non-secreted form of AFP (Hosokawa et al, 1989) could also lead to this result.

Studies by Hillaire et al (1994) have shown that hepatitis patients with positive detection of albumin mRNA in blood samples have poor prognosis. Whether the presence of AFP mRNA-expressing cells in some hepatitis patients, as found in the present study, indicates the presence of early-stage HCC tumours and poor prognosis remains to be defined. The presence of AFP mRNA in blood may not specifically indicate the presence of HCC cells. Therefore, identification of tumour-specific genes for hepatoma is very important for the molecular diagnosis of the haematogenous metastasis of hepatoma cells.

The AFP gene is a tumour-associated gene. Most normal tissues do not express AFP. It is primarily expressed in liver cells, as well as in the gut, stomach, trophoblast, lung or pancreas and to a lesser extent in the fetus and newborn infant (Shi et al, 1985; Nahon et al, 1988). It is not known whether AFP is expressed by the haematopoietic cells in fetal blood. However, Esteban et al (1993) have shown by *in situ* hybridization that activated monocytes and lymphocytes from adults express AFP mRNA. The positive AFP mRNA expression detected in all five samples of umbilical cord blood may have been caused by the expression of AFP mRNA by peripheral mononuclear cells or by trophoblast cells that were shed

© Cancer Research Campaign 1997

in the blood during delivery. The blood barrier in the placenta that separates the blood circulation of the fetus and mother is incomplete. Some molecules, viruses and cells can cross the barrier and circulate in the blood of the mother and fetus (Adkison et al, 1994). This may explain why all five blood samples from pregnant women had positive AFP mRNA expression.

In summary, we have described a PCR-based technique that can detect circulating hepatoma cells or hepatocytes in patients with HCC or hepatitis. This method may detect extra-hepatic metastatic tumour cells in the blood earlier than traditional imaging methods in HCC patients. Because of the expression of AFP by non-tumour cells and the potential presence of circulating AFP-expressing hepatocytes in hepatitis patients, the use of HCC-specific marker genes in combination with nested PCR is necessary to provide an early, sensitive and specific diagnostic method for the clinical staging of HCC.

### ACKNOWLEDGEMENTS

The authors thank Dr Cheng-Po Hu for providing Hep 3B cells, Dr Jah-Yao Liu and Mu-Hsien Yu for collecting samples and Ms Lily Chao for technical assistance.

This study was supported in part from the National Science Council (NSC83-0412-B016-052, NSC83-0412-B016-053), the Department of Health DOH-83-HR-203 and Academia Sinica, Republic of China.

### REFERENCES

Adkison LR, Andrews RH, Vowell NL and Koontz WL (1994) Improved detection of fetal cells from maternal blood with polymerase chain reaction. *Am J Obstet Gynecol* **170**: 952–955

Badley JE, Bishop GA, St John T and Frelinger JA (1988) A simple, rapid method for the purification of poly A+ RNA. *Biotechniques* **6**: 114–116

Bellet DH, Wands JR, Isselbacher KJ and Bohuon C (1985) Serum alpha fetoprotein levels in human disease: perspective from a highly specific monoclonal radioimmunoassay. *Proc Natl Acad Sci USA* **81**: 3869–3873

Chen DS (1987) Hepatitis B virus infection, its sequelae, and prevention in Taiwan. In *Neoplasms of the Liver*, Okuda K and Ishak KG (eds) pp. 71–80. Springer-Verlag: Tokyo

Deguchi T, Doi T, Ehara H, Ito S, Takahashi Y, Nishino Y, Fujihiro S, Kawamura T, Komeda H, Horie M, Kaji H, Shimokawa K, Tanaka T and Kawada Y (1993) Detection of micrometastatic prostate cancer cells in lymph nodes by reverse transcriptase–polymerase chain reaction. *Cancer Res* **53**: 5350–5354

Di Bisceglie AM and Hoofnagle JH (1989) Elevations in serum alpha-fetoprotein levels in patients with chronic hepatitis B. *Cancer* **64**: 2117–2120

Esteban C, Trojan J, Macho A, Mishal Z, Lafarge-Frayssinet CH and Uriel J (1993) Activation of an alpha-fetoprotein/receptor pathway in human normal and malignant peripheral blood mononuclear cells. *Leukemia* **7**: 1807–1816

Gibbs PE, Zielinski R, Boyd C and Dugaiczyk A (1987) Structure, polymorphism, and novel repeated DNA elements revealed by a complete sequence of the human α-fetoprotein gene. *Biochemistry* **26**: 1332–1343

Hillaire S, Barbu V, Boucher E, Moukhtar M and Poupon R (1994) Albumin messenger RNA as a marker of circulating hepatocytes in hepatocellular carcinoma. *Gastroenterology* **106**: 239–242

Hioki O, Watanabe A, Minemura M and Tsuchida T (1993) Chemical significance of serum hepatocyte growth factor levels in liver disease. *J Med* **24**: 35–46

Hosokawa S, Muramatsu M and Nagaike K (1989) Detection of membrane-bound α-fetoprotein in human hepatoma cell lines by monoclonal antibody 19F12. *Cancer Res* **49**: 361–366

Israeli RS, Miller WH JR, SU SL, Powell CT, Fair WR, Samadi DS, Huryk RF, Deblasio A, Edward ET, Wise GJ and Heston WDW (1994) Sensitive nested reverse transcription polymerase chain reaction detection of circulating prostatic tumour cells: comparison of prostate-specific membrane antigen and prostate-specific antigen-based assays. *Cancer Res* **54**: 6306–6310

Jiang WG, Hallett MB and Puntis MC (1993) Hepatocyte growth factor/scatter factor, liver regeneration and cancer metastasis. *Br J Surg* **80**: 1368–1373

Johnsum PWM, Burchill SA and Selby PJ (1995) The molecular detection of circulating tumour cells. *Br J Cancer* **72**: 268–276

Kar S and Carr BI (1995) Detection of liver cells in peripheral blood of patients with advanced-stage hepatocellular carcinoma. *Hepatology* **21**: 403–407

Knowles BB, Howe CC and Aden DP (1980) Human hepatocellular carcinoma cell lines that secret the major plasma proteins and hepatitis B surface antigens. *Science* **209**: 497–499

Komeda T, Fukuda Y, Sando T, Kita R, Furukawa M, Nishida N, Amenomori M and Nakao K (1995) Sensitive detection of circulating hepatocellular carcinoma cells in peripheral venous blood. *Cancer* **75**: 2214–2219

Lai CL, Gregory PB, WU PC, Lok AS, Wong KP and Ng MM (1987) Hepatocellular carcinoma in Chinese males and females: possible causes for the male predominance. *Cancer* **60**: 1107–1110

Liotta LA and Stetler-Stevenson WG (1991) Tumour invasion and metastasis: an imbalance of positive and negative regulation. *Cancer Res* **51**: 5054s–5059s

Matsumura Y and Tarin D (1992) Significance of CD44 gene products for cancer diagnosis and disease evaluation. *Lancet* **340**: 1053–1058

Matsumura M, Niwa Y, Kato N, Komatsu Y, Shina S, Kawabe T, Kawase T, Kawase T, Toyoshima H, Ihori M, Shiratori Y and Omata M (1994) Detection of α-fetoprotein mRNA, an indicator of hematogenous spreading hepatocellular carcinoma, in the circulation: a possible predictor of metastatic hepatocellular carcinoma. *Hepatology* **20**: 1418–1425

Matsumura M, Niwa Y, Hikiba Y, Okano KI, Kato N, Shiina S, Shiratori Y and Omata M (1995) Sensitive assay for detection of hepatocellular carcinoma asociated gene transcription (alpha-fetoprotein mRNA) in blood. *Biochem Biophys Res Commun* **207**: 813–818

Mattano La JR, Moss TJ and Emerson SG (1992) Sensitive detection of rare circulating neuroblastoma cells by the reverse transcriptase–polymerase chain reaction. *Cancer Res* **52**: 4701–4705

Moreno JG, Croce CM, Fischer R, Monne M, Vihko P, Mulholland SG and Gomella LG (1992) Detection of hematogenous micrometastasis in patients with prostate cancer. *Cancer Res* **52**: 6110–6112

Morinaga T, Sakai M, Wegmann TG and Tamaoki T (1983) Primary structures of human α-fetoprotein and its mRNA. *Cancer Res* **80**: 4604–4608

Nahon J-L, Tratner I, Poliard A, Presse F, Poiret M, Gal A and Sala-Trepat JM (1988) Albumin and α-fetoprotein gene expression in various nonhepatic rat tissues. *J Biol Chem* **263**: 11436–11442

Naito H, Kuzumaki N, Uchino J, Kobayashi R, Snikano T, Ishikawa Y and Matsumoto S (1991) Detection of tyrosine hydroxylase mRNA and minimal neuroblastoma cells by reverse transcription–polymerase chain reaction. *Eur J Cancer* **27**: 762–765

Pfleiderer C, Zoubek A, Gruber B, Kronberger M, Ambros PF, Lion T, Fink F-M, Gadner H and Kovar H (1994) Detection of tumour cells in peripheral blood and bone marrow from Ewing tumour patients by RT–PCR. *Int J Cancer* **64**: 135–139

Shi W-K, Hopkins B, Thompson S, Heath JK, Luke BM and Graham CF (1985) Synthesis of apoliproteins, alphafoetoprotein, albumin and transferrin by the human foetal yolk sack and other foetal organs. *J Embryol Exp Morph of* **85**: 191–206

Schoenfeld A, Luqmani Y, Smith D, O'Reilly S, Shousha S, Sinnett HD and Coombes RC (1994) Detection of breast cancer micrometastases in axillary lymph nodes by using polymerase chain reaction. *Cancer Res* **54**: 2986–2990

Silver HK, Deneault J, Gold P, Thompson WG, Shtster J and Freedman SO (1974) The detection of alpha-1-fetoprotein in patients with viral hepatitis. *Cancer* **34**: 244–247

Smith B, Selby P, Southgate J, Pittaman K, Bradley C and Blair GE (1991) Detection of melanoma cells in peripheral blood by means reverse transcriptase and polymerase chain reaction. *Lancet* **338**: 1227–1229

Stolz DB and Michalopoulos GK (1994) Comparative effects of hepatocyte growth factor and epidermal growth factor on motility, morphology, mitogenesis and signal transduction of primary rat hepatocytes. *J Cell Biochem* **55**: 445–464

Tso JY, Sun XH, Kao TH, Reece KS and Wu R (1985) Isolation and characterization of rat and human glyceraldehyde-3-phosphate dehydrogenase cDNAs: genomic complexity and molecular evolution of the gene. *Nucleic Acids Res* **13**: 2485–2502

© Cancer Research Campaign 1997